# Targeting Interleukin-6 Signaling with Reactive-Oxygen-Species-Responsive Hydrogel to Promote Regeneration after Spinal Cord Injury

**DOI:** 10.34133/bmr.0395

**Published:** 2026-07-22

**Authors:** Runlin Wen, Xinghui He, Kai Zhang, Wanrong Ma, Zhiquan Yang, Dingyang Liu, Ge Long, Xing Li

**Affiliations:** ^1^ Department of Biochemistry and Molecular Biology, College of Life Sciences, Central South University, Changsha 410078, China.; ^2^ Department of Neurosurgery, Xiangya Hospital of Central South University, Changsha 410008, China.; ^3^ Department of Anesthesia, The Third Xiangya Hospital of Central South University, Changsha 410013, China.

## Abstract

Spinal cord injury (SCI) triggers an excessive inflammatory response, characterized by the up-regulation of various inflammatory factors that impede neural regeneration and functional recovery. Interleukin-6 (IL-6) is an early and critical inflammatory mediator observed in lesions post-SCI. Antagonizing the signaling pathway presents a promising strategy to mitigate early inflammation and secondary injury after trauma. Here, we identified specific activation of the IL-6 receptor in neurons and microglia in lesions, indicating their responsiveness to early up-regulated IL-6 signaling within the microenvironment. In vitro, neutralizing IL-6 signaling in microglia effectively alleviated their inhibitory effects on neuronal axon growth in conditioned media. Building on this, we developed a reactive-oxygen-species-responsive hydrogel for the sustained local delivery of tocilizumab, an IL-6 receptor antagonist, and implanted it in a complete transection SCI model. In vivo, sustained IL-6 receptor blockade effectively reduced early inflammatory cell infiltration, modulated microglial polarization toward an anti-inflammatory phenotype, and fostered neuronal regeneration within the lesion. Importantly, this therapeutic intervention promoted long-term hind limb functional recovery in SCI mice. This study underscores the therapeutic potential of precisely targeting early inflammatory cytokine signaling pathways, particularly IL-6, to improve outcomes after SCI.

## Introduction

Spinal cord injury (SCI) is a severe condition in the central nervous system, resulting in paralysis and sensory loss below the lesion [1]. The initial trauma leads to cellular destruction and vascular rupture, along with an increase in cytokine expression and immune cell infiltration. These changes finally trigger excessive neuroinflammation, contributing to additional cell death [2]. Modulating the excessive inflammatory response to protect tissue and function is the main goal for neuroprotective therapy [3].

Interleukin-6 (IL-6) is 1 of the 3 earliest up-regulated inflammatory cytokines following SCI [2,4]. The expression increases rapidly in the early phase and declines to baseline levels within a week postinjury [5]. Previous studies indicated that IL-6 may participate in acute inflammatory responses, reactive gliosis, and neurodegenerative processes [6–11]. Researchers employed the IL-6 receptor (IL-6R) monoclonal antibody in SCI and observed that blocking the IL-6 signaling pathway suppresses macrophage activation and enhances motor functional recovery [6]. Surprisingly, some studies demonstrated that IL-6 exerted beneficial effects on axonal regeneration and locomotor function recovery, highlighting the dual role of this cytokine in SCI repair [12,13]. However, these studies analyzed therapeutic outcomes solely through intravenous or intrathecal administration, which differed from localized drug delivery. Targeted local delivery to the lesion is essential for demonstrating the precise role of IL-6 in SCI repair.

In this study, we identified the sources of IL-6 and the corresponding receptor expression patterns postinjury and prepared an injectable hydrogel composed of quaternized chitosan (QCS) and tannic acid (TA), loaded with tocilizumab, which could respond to inflammatory stimulation and enable localized sustained drug release. We found that local administration of tocilizumab reduced immune cell infiltration and modulated glial cell polarization toward an anti-inflammatory phenotype, indicating that blocking IL-6R can alleviate the inflammatory response and shift the overall immune profile toward an anti-inflammatory state. Based on the improved inflammatory microenvironment, we further examined the level of neural regeneration within the lesion and found that tocilizumab treatment promoted the regrowth of functional nerve fibers and decreased the deposition of chondroitin sulfate proteoglycans. These beneficial effects persisted for 2 months and provided support for hind limb functional recovery in mice. Taken together, after SCI, neurons and microglia are the earliest responders to elevated IL-6 in the acute phase. Local application of a tocilizumab-loaded sustained-release hydrogel to neutralize IL-6 signaling in the injured area reduces immune cell infiltration and alleviates the immune response while promoting axonal regeneration and synapse formation, ultimately improving the prognosis in SCI mice.

## Materials and Methods

### Animal

The male C57BL/6J mice (6 to 8 weeks old) used in this study were supplied by the Animal Experiment Center of Central South University (Changsha, China). All mice were maintained under standard specific-pathogen-free conditions with a regular light/dark cycle. During the whole experiment, food and water were available. All animal experiments were reviewed and approved by the Animal Ethics Committee of Xiangya Hospital, Central South University. The whole procedure was performed in compliance with the National Institutes of Health Guide for the Care and Use of Laboratory Animals. For the isolation of primary neurons, neonatal C57BL/6J mice (1 to 2 d old) were supplied by SLAC Animal Corporation (Changsha, China).

### Surgery process and treatment

Mice were anesthetized with an intraperitoneal injection of sodium pentobarbital (100 mg/kg) during surgery. Following hair removal, a midline skin incision of approximately 1 cm was made to expose the T7 to T10 vertebrae. A precise 2-mm laminectomy was carried out at the T8 vertebral level under microscopic guidance, and a 2-mm portion of the spinal cord was removed. The lesion was gently washed with sterile saline, and bleeding was treated with gelatin sponge. In the treatment group (Toc+Gel), a reactive oxygen species (ROS)-responsive hydrogel containing 400 μg/ml tocilizumab (anti-IL-6R, Cat. A2012, Selleckchem.com) was injected into the lesion cavity using a syringe, matching the size of the removed tissue. In 2 control groups (Gel and Control), either hydrogel without antibody or no treatment was administered. The skin was then sutured in layers, and all animals were placed back in their cages for postoperative recovery.

### ROS-responsive hydrogel preparation

To prepare the ROS-responsive QCS–TA hydrogel, 3 g of QCS was first dissolved in 3 ml of deionized water, followed by the addition of 2.5 mg of antibody. The mixture was stirred thoroughly. Then, a solution of TA and dilute hydrochloric acid (mixed at a 1:30 ratio) was added to the QCS solution and stirred evenly. Subsequently, 100 μl of sodium bicarbonate was added while rapidly stirring the mixture for approximately 60 s, resulting in the formation of a QCS–TA ROS-responsive hydrogel loaded with antibody.

### Tissue processing and immunostaining

Spinal cord tissues from the 3 groups were collected at multiple time points (1, 3, 7, and 60 days postinjury [dpi]). The brief procedure was as follows: mice were transcardially perfused with 4% paraformaldehyde, after which the spinal cords were carefully dissected and collected. The tissues were postfixed in 4% paraformaldehyde at 4 °C overnight and then transferred to a 20% sucrose solution at 4 °C for 48 h, followed by incubation in 30% sucrose solution for 72 h. After embedding in optimal cutting temperature compound (Cat. AC28L458, Life-iLab, Shanghai, China), the spinal cord tissues were sectioned at a 15-μm thickness along the dorsoventral axis using a freezing microtome (RWD Life Science, Shenzhen, China).

Immunofluorescence staining was conducted as follows: frozen sections were first equilibrated to room temperature and subsequently washed 3 times in phosphate-buffered saline (PBS) for 5 min each. Permeabilization was performed using 0.5% Triton X-100 for 20 min, followed by 3 additional PBS washes. Sections were blocked with 5% normal donkey serum (Cat. SL050, Beijing Solarbio Science & Technology Co., Ltd.) for 1 h to block nonspecific binding, after removing the blocking solution, the sections were incubated overnight at 4 °C with primary antibodies, including Iba-1 (MA5-27726, 1:500; Invitrogen), IL-6 (ab290735, 1:100; Abcam), IL-6R (23457-1-Ig, 1:500; Proteintech), CD206 (ab64693, 1:500; Abcam), beta III tubulin (Tuj-1; ab18207, 1:500; Abcam), microtubule-associated protein 2 (Map-2; ab32454, 1:500; Abcam), glial fibrillary acidic protein (14-9892-82, 1:500; Invitrogen), Olig2 (sc-293163, 1:100; Santa Cruz), vimentin (60330-1-Ig, 1:100; Proteintech), Ly6G (NBP2-00441, 1:200; Bio-Techne), inducible nitric oxide synthase (iNOS; 14592082, 1:100; Invitrogen), F4/80 (30325T, 1:200; CST), vesicular glutamate transporter 2 (vGlut2; 29209-1-AP, 1:500; Proteintech), glutamic acid decarboxylase 67 (GAD67; 67648-1-Ig, 1:500; Proteintech), and CS56 (C8035, 1:1,000; Sigma).

After overnight incubation, sections were washed 3 times with PBS (5 min each), followed by a 2-h incubation with secondary antibodies at room temperature. These included Alexa Fluor 488-conjugated donkey anti-mouse antibody (A21202, Invitrogen), Alexa Fluor 488-conjugated donkey anti-rabbit antibody (A21203, Invitrogen), and Alexa Fluor 568-conjugated donkey anti-rabbit antibody (A10042, Invitrogen). After secondary incubation, sections were washed again with PBS. Excess moisture was gently removed, and sections were sealed with an antifade sealer containing 4′,6-diamidino-2-phenylindole. Fluorescence images were taken by a fluorescence microscope (Zeiss Imager M2, Germany).

### Primary neuron extraction

Fresh spinal cord tissues were dissected from neonatal mice in 5 min and immediately immersed in high-glucose Dulbecco’s modified Eagle medium (DMEM). After the meninges were carefully removed, the tissues were finely chopped with ophthalmic scissors and digested with 0.25% trypsin at 37 °C for 20 min, with gentle pipetting every 10 min. A trypsin inhibitor was then added to terminate the digestion. The tissue suspension was filtered through a 100-μm cell strainer (Biosharp) to generate a single-cell suspension and then centrifuged at 500 × g for 5 min. The cell pellet was resuspended and seeded into 6-well plates (Corning, USA) at a density of 2 × 10^6^ cells per well. Neurons are cultured in Neurobasal medium (Invitrogen, USA) containing 2% B27 supplement, 30% glucose (Sigma, USA), 1% glutamine (Invitrogen, USA), and 1% penicillin/streptomycin (Invitrogen, USA). After 5 d of culture, the neurons exhibited robust axonal outgrowth. In in vitro experiments, the concentration of tocilizumab preadded to the neuronal culture medium was 0.6 μg/μl.

### BV2 cell culture

The BV2 cell line used in this study was purchased from Procell Life Science and Technology Corporation. The cell culture process was as follows: BV2 cells were cultured in complete medium based on DMEM, which contained 10% fetal bovine serum (Cat. 164210, Wuhan Procell Biotechnology Co., Ltd.) and 1% penicillin/streptomycin (Gibco, NY, USA), and incubated at 37 °C with 5% CO_2_. For the 6-well plates that required intervention, 80 ng/ml of IL-6 (Cat. HY-P78853, MedChemExpress, USA) was added to the culture medium for 24 h. Thereafter, half of the culture medium was aspirated from each well and mixed with fresh neuronal Neurobasal medium at a 1:1 ratio to serve as conditioned medium for primary neurons.

### Behavioral assessment

The study used the Basso Mouse Scale (BMS) score to assess hind limb motor function after SCI in mice, which were placed in an open field. Two researchers who were blinded to the experimental information of animals recorded the BMS score for each mouse. The detailed rules of the BMS scoring system were described previously [14].

### RNA-seq and bioinformatics analysis

Tissue segments (1 mm) were isolated from mice, and total RNA was extracted using TRIzol (Invitrogen, USA) solution. High-throughput RNA sequencing (RNA-seq) was conducted by LC Bio Technology Corporation (Hangzhou, China). After obtaining the total RNA from the samples, the quantity and purity of the RNA were analyzed using Agilent 5300 Fragment Analyzer (M5311AA, Agilent, CA, USA), and high-quality RNA samples (with RNA integrity number > 7.0) were selected for library construction. After RNA extraction, messenger RNA (mRNA) was purified from the total RNA in 2 rounds. Following purification, the mRNA was fragmented (Yeasen Cat. 12340ES97, China), and cleaved DNA was synthesized by reverse transcription. Finally, 2× 150-bp paired-end sequencing (PE150) was performed on Illumina NovaSeq X Plus.

Gene differential expression analysis was performed using the DESeq2 software. Differentially expressed genes (DEGs) were defined as those with a false discovery rate less than 0.05 and an absolute fold change ≥2. Enrichment analysis of Gene Ontology (GO) functions and Kyoto Encyclopedia of Genes and Genomes (KEGG) pathways was then conducted on the DEGs.

### Statistical analysis

Data are reported as mean ± standard error of the mean. All statistical analyses were conducted in GraphPad Prism 9.5.1 (733). For comparisons involving more than 2 groups, a one-way analysis of variance was applied followed by Tukey’s test to account for multiple comparisons. Two-group differences were evaluated with an unpaired *t* test. *P* < 0.05 was considered statistically significant.

## Results

### IL-6 mainly targets microglia and neurons after SCI

**Fig. 1.**
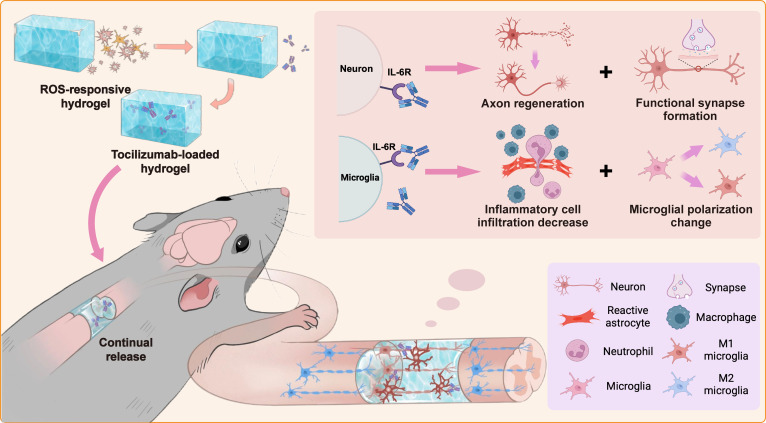
Schematic illustration of tocilizumab-loaded reactive oxygen species (ROS)-responsive hydrogel for spinal cord injury (SCI) treatment. Local application of a tocilizumab-loaded hydrogel neutralizes interleukin-6 (IL-6) signaling in lesions, reducing immune infiltration and inflammation while promoting axonal regeneration and synapse formation, which improves prognosis in SCI mice. IL-6R, IL-6 receptor.

**Fig. 2.**
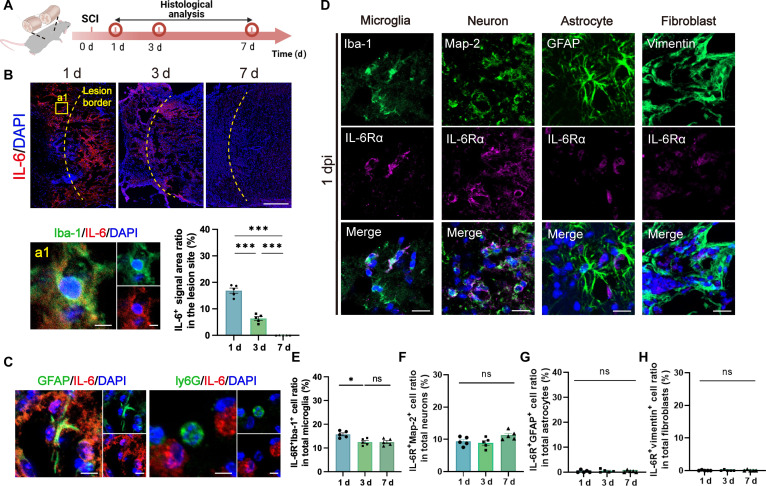
The main source of interleukin-6 (IL-6) and expression pattern of the IL-6 receptor (IL-6R). (A) Time point selection of spinal cord injury and histological analysis. (B) Percentage of IL-6^+^ area of lesion site in control groups at 1, 3, and 7 days postinjury (dpi) (*n* = 5 images). Scale bar = 200 μm. (C) The expression of IL-6 in astrocytes and neutrophils at 1 dpi. Scale bar = 10 μm. (D) The profile of IL-6R expression in 4 cell populations at 1 dpi. Scale bar = 20 μm. (E to H) The proportions of IL-6R^+^ cells among 4 cell populations at 1 dpi (*n* = 5 images). Error bars represent the standard error. The comparisons between multiple groups were conducted using one-way analysis of variance (ANOVA) with Tukey’s test. *P < 0.05; ***P < 0.001; ns, not significant.

First, we established a complete transection SCI model and examined IL-6 expression levels in lesions and the margin area at different time points postinjury (Figs. 1 and 2A). Consistent with previous studies, IL-6 levels increased rapidly within 24 h after injury, with the IL-6-positive area accounting for 16.865% ± 0.982% per field, followed by a gradual decline to 6.354% ± 0.696% (*P* < 0.001) [2]. By day 7 postinjury, IL-6 expression was nearly undetectable in lesions (Fig. 2B). Further analysis of IL-6 sources revealed that IL-6 was primarily derived from microglia and astrocytes (Fig. 2B and C). Subsequently, we explored the cellular populations that IL-6 may affect. During the whole period of IL-6 expression, the corresponding receptor (IL-6R) was detected only in microglia and neurons, suggesting that IL-6 mainly targets these 2 cell types (Fig. 2D to H and Fig. S1).

### Tocilizumab counteracts excessive IL-6 to promote axonal regeneration in vitro

**Fig. 3.**
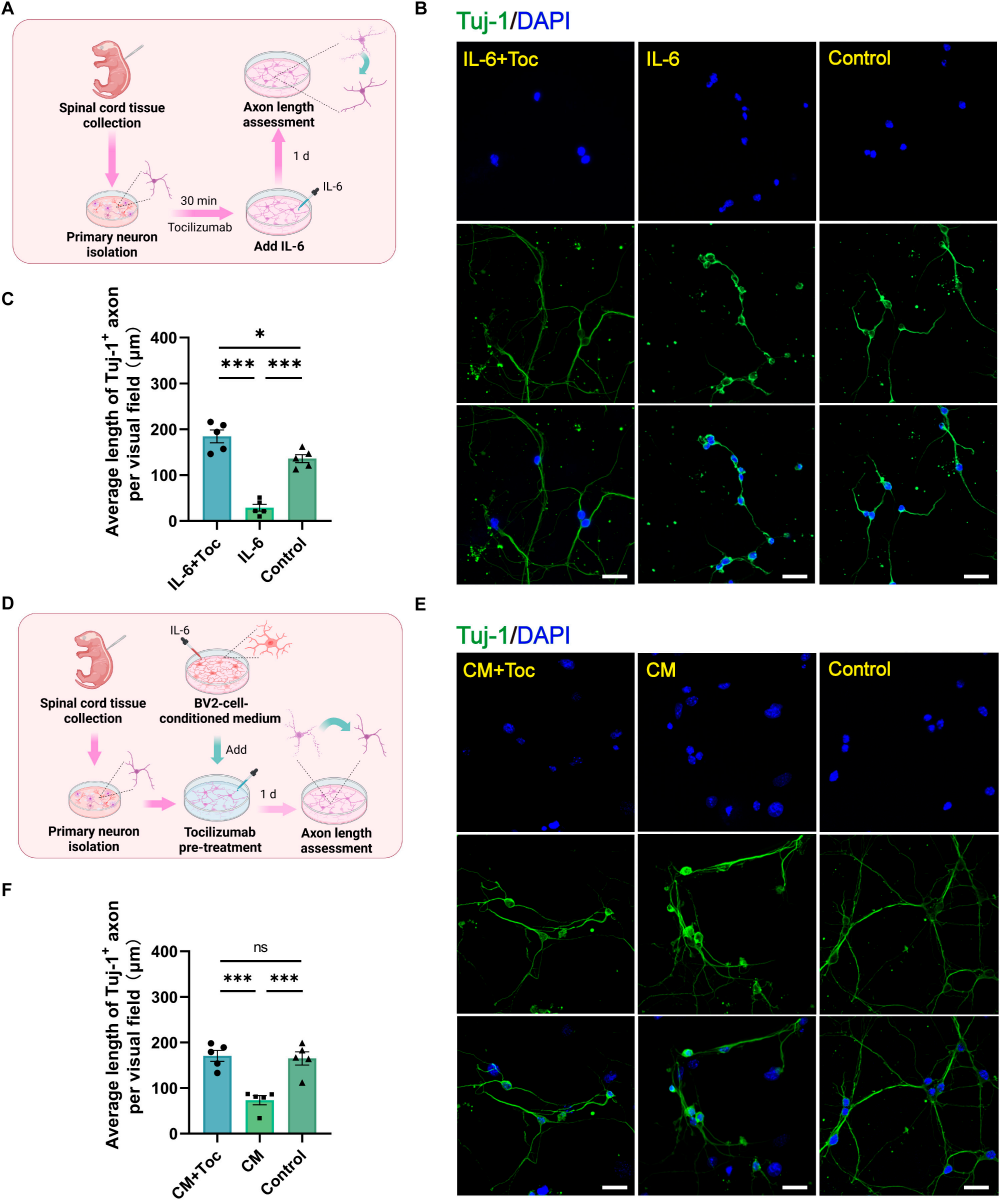
Tocilizumab improves early axon regeneration in vitro. (A) The isolation of primary neuron and process of the first in vitro experiment. (B) The profile of Tuj-1^+^ axons’ length in 3 groups. Scale bar = 20 μm. (C) The statistical analysis of the average axon length per visual field across the 3 groups (*n* = 5 images). (D) The isolation of primary neuron and process of the second in vitro experiment. (E) The profile of Tuj-1^+^ axons’ length in 3 groups. Scale bar = 20 μm. (F) The statistical analysis of the average axon length per visual field in the CM+Toc, CM, and Control groups (*n* = 5 images). *P < 0.05; ***P < 0.001. CM, conditioned medium; Toc, tocilizumab; IL-6, interleukin-6; Tuj-1, beta III tubulin.

After observing the dynamic changes of this cytokine, we aim to assess the impact of transient excessive IL-6 on host neural function, particularly neuronal responses. Therefore, we established an in vitro neuronal culture system and examined the effects of IL-6 and its receptor blockade with tocilizumab. We first performed microsurgical dissection and enzymatic digestion of spinal cords from P1 neonatal mice to extract neurons, which were cultured in vitro. In the treatment group, neurons were pre-treated with tocilizumab for 30 min before IL-6 administration (Fig. 3A). The 2 control groups either received IL-6 alone or remained untreated.

Meanwhile, to simulate the indirect effect of IL-6, neurons were treated with conditioned media obtained from IL-6-treated BV2 cells, and 3 experimental groups were established. After 12 h of culture, we assessed axonal regeneration (Fig. 3D). The results showed that IL-6 treatment alone reduced average axon length to approximately one-fourth of the untreated group (IL-6: 29.42 ± 7.323 μm; Control: 136.44 ± 8.798 μm; Fig. 3B and C).

However, pre-treatment with tocilizumab effectively promoted axon growth, exceeding the axon length of 2 control groups (Toc+IL-6: 184.81 ± 14.121 μm; *P* (Toc+IL-6 vs IL-6) < 0.001, *P* (Toc+IL-6 vs Control) = 0.02; Fig. 3B and C). A similar regenerative effect was observed in the experiment investigating the indirect effects of IL-6. Neurons treated with BV2-conditioned medium (pre-treated with IL-6) showed axonal retraction (CM: 79.913 ± 4.025 μm; Control: 167.27 ± 13.081 μm; *P* (CM+Toc vs CM) < 0.001, *P* (CM vs Control) < 0.001; Fig. 3E and F), whereas tocilizumab pre-treatment restored axon length to levels comparable to those in the untreated group (CM+Toc: 170.97 ± 11.957 μm; *P* (CM+Toc vs Control) = 0.97; Fig. 3E and F).

### The ROS-responsive hydrogel-based delivery system achieves stable tocilizumab release

**Fig. 4.**
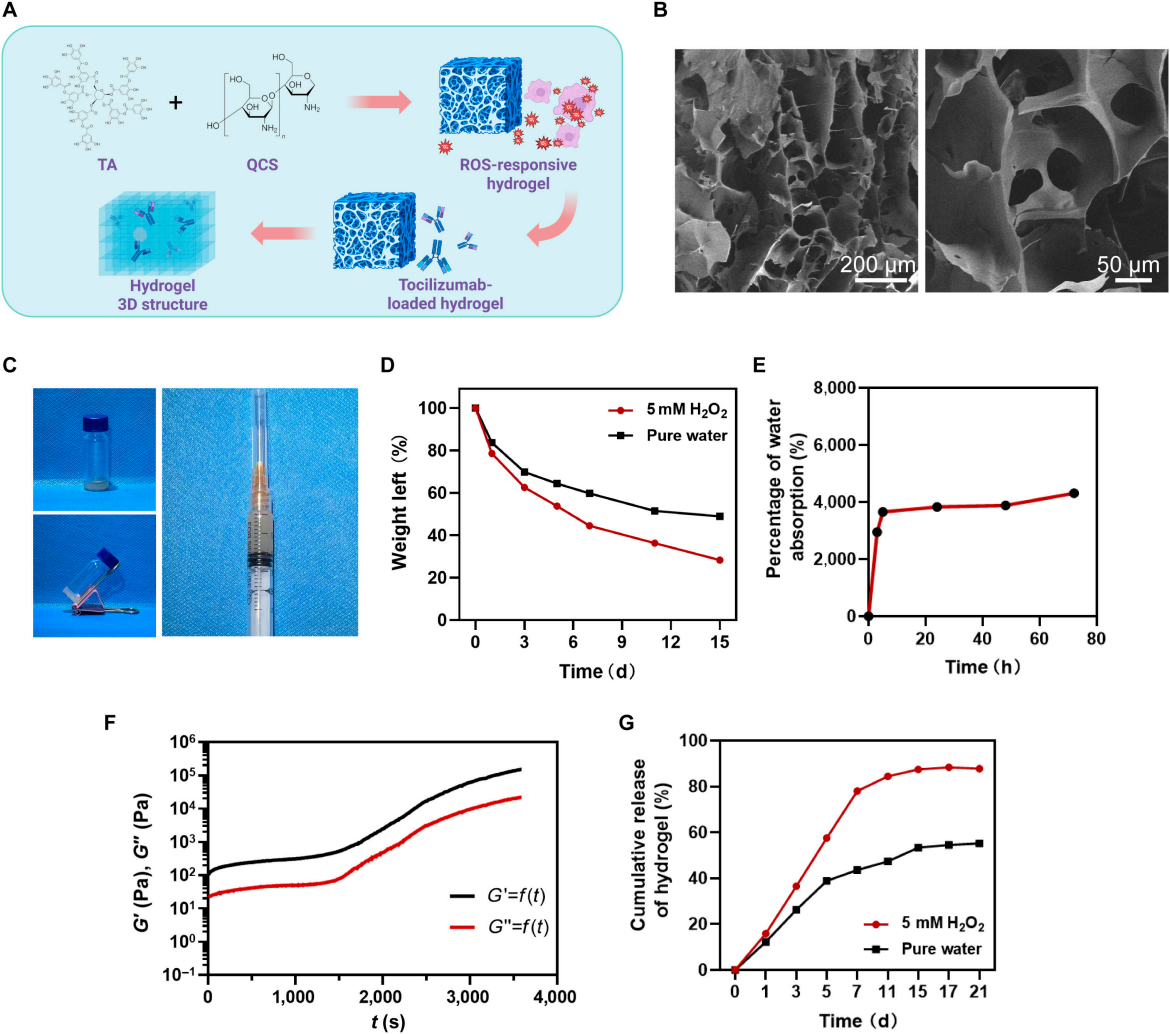
The construction of a loaded reactive oxygen species (ROS)-responsive hydrogel and its characteristics. (A) The preparation process of the ROS-responsive hydrogel. (B) Morphological characteristics of the hydrogel observed under electron microscopy. (C) The ROS-responsive hydrogel exhibits good adhesiveness and injectability. (D) Degradation rate of the hydrogel under inflammatory conditions in vitro. (E) Water absorption capacity of the hydrogel in vitro. (F) Rheological characteristics of the ROS-responsive hydrogel. (G) Cumulative release of tocilizumab from the hydrogel under 2 different conditions in vitro. TA, tannic acid; QCS, quaternized chitosan.

Previous in vitro experiments have demonstrated that tocilizumab promotes axonal regeneration; whether this beneficial effect can be translated in vivo remains unclear. However, systemic intravenous administration resulted in insufficient drug accumulation at the lesion site. To achieve localized drug delivery, we first developed an ROS-responsive hydrogel capable of sustained drug release and loaded it with tocilizumab, an IL-6R antagonist (Figs. 4A and 1). We then evaluated the hydrogel’s degradation rate, pore size, water absorption capacity, and rheological properties and found that it exhibited excellent adhesiveness and injectability (Fig. 4B to F). Under in vitro conditions, the hydrogel demonstrated rapid degradation and released approximately 90% of the encapsulated drug within 15 d (Fig. 4G).

### Tocilizumab promotes the anti-inflammatory polarization of microglia in lesions

**Fig. 5.**
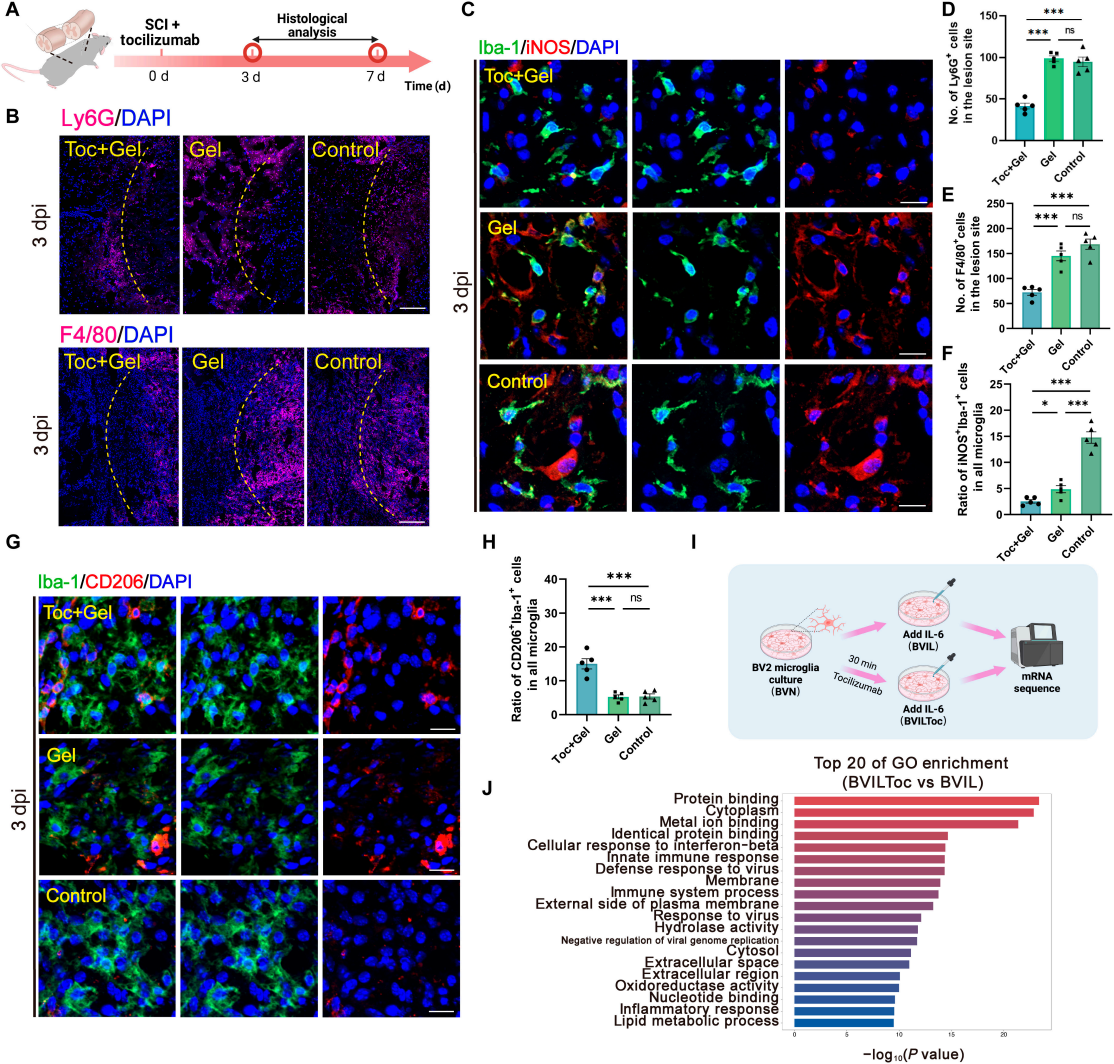
The administration of tocilizumab improves inflammatory microenvironment in lesions. (A) Time point selection of spinal cord injury and histological analysis. (B) Infiltration of Ly6G^+^ neutrophils in the surrounding area. Scale bar = 200 μm. (C) The expression profile of inducible nitric oxide synthase (iNOS) in microglia within the lesion area. Scale bar = 10 μm. (D) The number of Ly6G^+^ neutrophils per field in the lesion (*n* = 5 images). (E) The number of F4/80^+^ macrophages per visual field in lesions. (F) The proportion of microglia expressing iNOS within the lesion area. Scale bar = 10 μm (*n* = 5 images). (G) The expression profile of iNOS^+^CD206^+^ cells in all microglia per field (*n* = 5 images). (H) The percentage of microglia expressing CD206 per visual field (*n* = 5 images). (I) The culture of BV2 microglia and process of in vitro experiment. (J) The top 20 Gene Ontology (GO) terms obtained from the analysis of differentially expressed genes (DEGs) between the BVILToc and the BVIL group.*P < 0.05; ***P < 0.001.

After preparing the hydrogel for localized sustained drug release, we divided the mice into 3 groups (Toc+Gel, Gel, and Control). At 3 dpi, we evaluated the infiltration of 2 types of immune cells, neutrophils and macrophages, in the lesion and surrounding areas (Fig. 5A). The results showed that in the 2 control groups without antibody treatment, nearly 100 neutrophils were observed per field within the lesion (Gel: 98.807 ± 3.169; Control: 94.801 ± 5.643). However, Toc+Gel exhibited fewer infiltrating neutrophils, with 41.60 ± 3.44 cells per field in the lesion (*P* (Toc+Gel vs Gel) < 0.001, *P* (Toc+Gel vs Control) < 0.001; Fig. 5B and D). In addition, we analyzed macrophage infiltration in the margin of lesion. Similar to the results observed for neutrophils, mice treated with the antibody showed markedly reduced macrophage infiltration, with 72.2 ± 5.757 F4/80^+^ macrophages per field (Fig. 5B and E). In contrast, the number of macrophages in the 2 control groups was more than twice that of the treated group (Gel: 145.2 ± 9.912; Control: 168.6 ± 10.1; *P* (Toc+Gel vs Gel) < 0.001, *P* (Toc+Gel vs Control) < 0.001; Fig. 5B and E).

These findings from the immune cells reveal that local treatment with tocilizumab attenuates the inflammatory response in lesions. Considering that resident microglia are not only key participants in the post-SCI inflammatory cascade but also the target of IL-6, we further examined the activation state of microglia and noticed a shift in microglial polarization. The proportion of iNOS^+^ microglia, indicative of the proinflammatory M1 phenotype, was decreased in the treated group, with iNOS^+^ area coverage around 1.5% per field (1.337% ± 0.31%), much lower than in the control groups (*P* (Toc+Gel vs Gel) = 0.02, *P* (Toc+Gel vs Control) < 0.001; Fig. 5C and F). In contrast, the area occupied by M2 phenotype microglia, characterized by anti-inflammatory properties, was nearly 3 times higher in the antibody-treated group compared to that in controls (Fig. 5G and H). Moreover, we also established an in vitro BV2 microglia system and performed bulk RNA-seq analysis on groups with or without tocilizumab treatment (Fig. 5I). The DEGs between the BVILToc and BVIL groups were primarily enriched in the GO terms associated with inflammatory response and innate immune response (Fig. 5J and Fig. S2). These results suggest that local application of tocilizumab improves the inflammatory microenvironment within the lesion, promoting a shift from a proinflammatory to an anti-inflammatory response.

### Tocilizumab promotes early neural regeneration in vivo

**Fig. 6.**
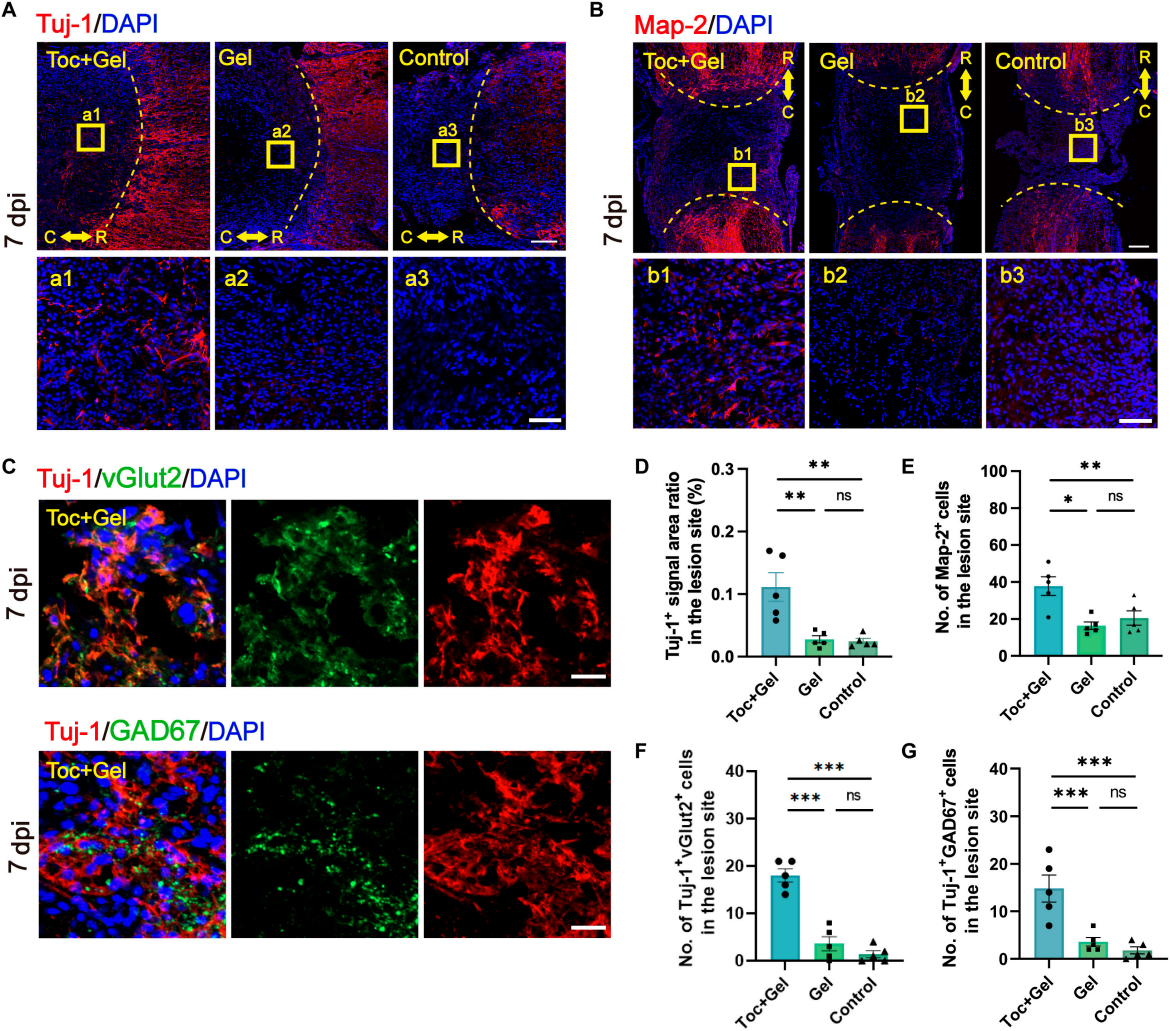
Tocilizumab improves early neural regeneration in lesions. (A and B) The profile of Tuj-1^+^ axons and Map-2^+^ neurons in 3 groups at 7 dpi. The short scale bar represents 200 μm, and the long scale bar represents 50 μm. (C) The colocalization level of Tuj-1^+^ axons with excitatory (vesicular glutamate transporter 2 [vGlut2]) or inhibitory (glutamic acid decarboxylase 67 [GAD67]) neurotransmitter markers. Scale bar = 20 μm. (D) The ratio of Tuj-1^+^ axons’ area per field in the lesion (*n* = 5 images). (E) The number of Map-2^+^ neurons per field in the lesion (*n* = 5 images). (F) The expression profile of Tuj-1^+^ axons colabeled with vGlut2 in the lesion (*n* = 5 images). (G) The expression profile of Tuj-1^+^ axons colabeled with GAD67 in the lesion (*n* = 5 images). *P < 0.05; **P < 0.01; ***P < 0.001. Tuj-1, beta III tubulin; Map-2, microtubule-associated protein 2‌.

Following the change of inflammatory response in lesions, we aimed to analyze how the improved inflammatory microenvironment affects the lesion. Due to the close relationship between the microenvironment and endogenous neural regeneration, we assessed the profile of neural regeneration in lesions at 7 dpi. Immunofluorescence staining revealed partial neural regeneration in the antibody-treated group; the area of Tuj-1^+^ axons accounted for 0.111% ± 0.022% of the lesion area, while the ratio of the positive area in both control groups was less than 1/1,000 (Gel: 0.027% ± 0.005%; Control: 0.024% ± 0.004%; *P* (Toc+Gel vs Gel) = 0.003, *P* (Toc+Gel vs Control) = 0.002; Fig. 6A and D). We observed that the regenerated axons were positive for either GAD67 or vGlut2 (Fig. 6C), which are markers for inhibitory and excitatory neurotransmitters, respectively. The average number of vGlut2^+^Tuj-1^+^ axons was 18.31 ± 1.378, and that of GAD67^+^Tuj-1^+^ axons was 14.80 ± 2.835 per field (Fig. 6F and G). Similarly, consistent with the former staining results, the number of mature neurons (Map-2^+^) in the lesion area increased in the Toc+Gel group (Fig. 6B). There were nearly 40 mature neurons per field of view (Toc+Gel: 37.80 ± 5.014), approximately twice the number observed in the 2 control groups (Gel: 16.40 ± 2.064; Control: 20.60 ± 3.855; *P* (Toc+Gel vs Gel) = 0.0052, *P* (Toc+Gel vs Control) = 0.0206; Fig. 6E).

### Tocilizumab promotes functional synapse formation in vitro

**Fig. 7.**
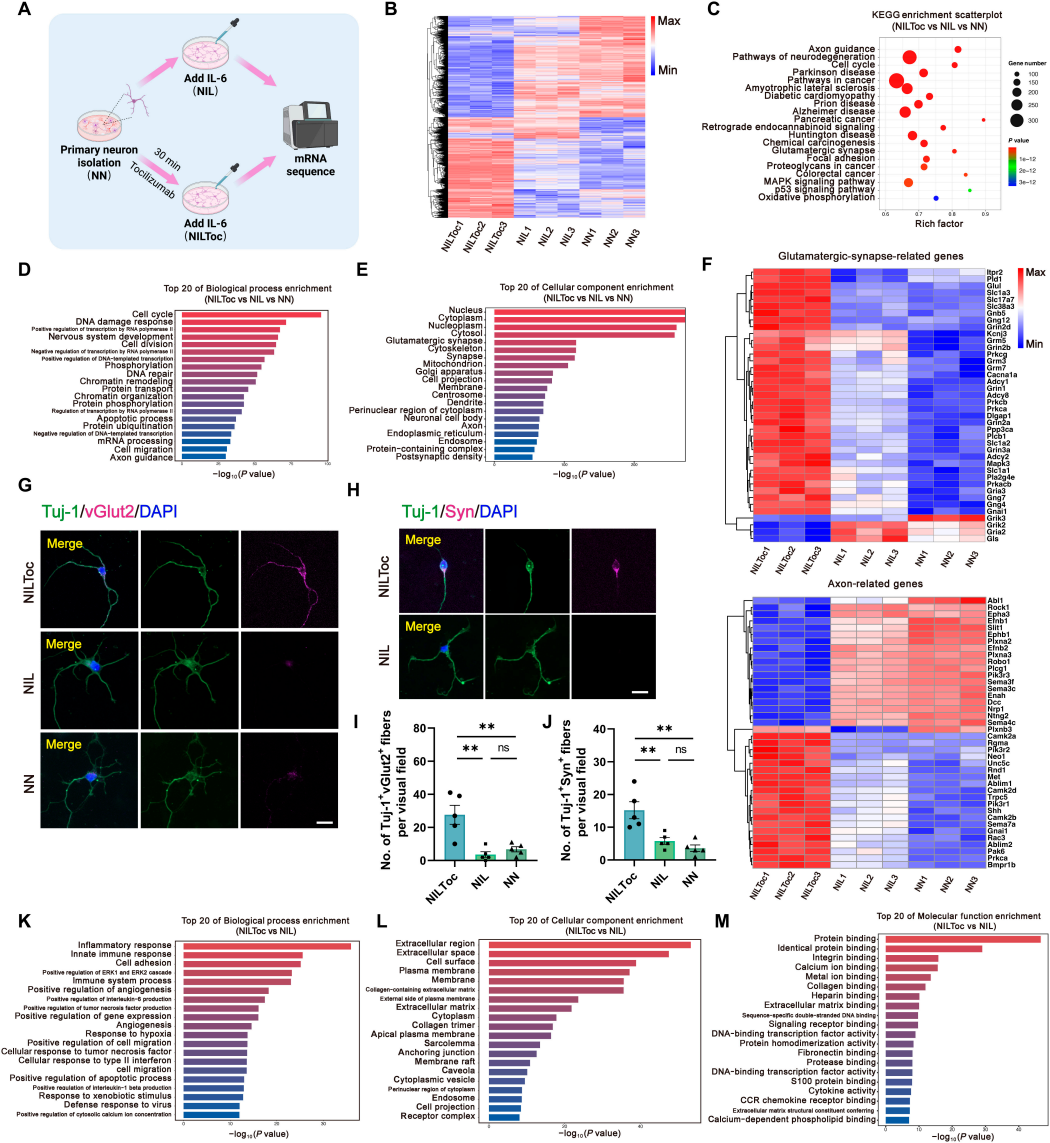
Targeting excessive interleukin-6 (IL-6) promotes functional synapse formation in vitro. (A) The isolation of primary neurons and sequencing process. (B) Heatmap of differentially expressed genes (DEGs) among 3 groups. (C) Kyoto Encyclopedia of Genes and Genomes (KEGG) pathway analysis of DEGs among 3 groups. (D and E) Gene Ontology (GO) enrichment analysis among 3 groups. (F) Heatmaps of the top 40 DEGs associated with glutamatergic synapse and axon GO terms. (G) The profile of Tuj-1^+^vGlut2^+^ axons among 3 groups. Scale bar = 20 μm. (H) The profile of synapse formation in cultured neurons between the NILToc and NIL groups. Scale bar = 20 μm. (I and J) Quantitative analysis of Tuj-1^+^vGlut2^+^ axons and Tuj-1^+^Syn^+^ axons among the 3 groups (*n* = 5 images). (K to M) Top 20 GO enrichment terms across 3 categories between the NILToc and NIL groups. vGlut2, vesicular glutamate transporter 2. **P < 0.01. Tuj-1, beta III tubulin.

The above in vivo and in vitro findings showed that early targeting of IL-6 signaling promoted functional axonal regeneration. To further investigate the transcriptomic changes induced by tocilizumab in neurons, we performed RNA-seq on neurons subjected to different interventions (Fig. 7A). The results showed that excessive IL-6 altered the transcriptional profiles of a portion of genes. In contrast, neurons treated with the antibody displayed markedly distinct transcriptional patterns compared with the 2 control groups (Fig. 7B and Fig. S3). The DEGs were subjected to GO and KEGG enrichment analyses, revealing potential associations with axon guidance and glutamatergic synapse formation (Fig. 7C to F and Fig. S4). Accordingly, we examined these 2 indicators across the 3 groups. Immunofluorescence results showed that excessive IL-6 did not alter the levels of neuronal axon glutamatergic synapse formation. In contrast, antibody-mediated inhibition of IL-6 signaling substantially promoted the formation of functional axons and synapses (Fig. 7G to J and Fig. S5). We then further analyzed differences before and after antibody treatment and found that the DEGs were enriched in GO terms related to the extracellular matrix and inflammatory responses (Fig. 7K to M and Fig. S6). Collectively, these results indicated that tocilizumab promoted the formation of functional synapses in cultured neurons and regulated inflammation-associated gene expression within neurons.

### Tocilizumab supports long-term axonal growth and functional restoration of hind limbs

**Fig. 8.**
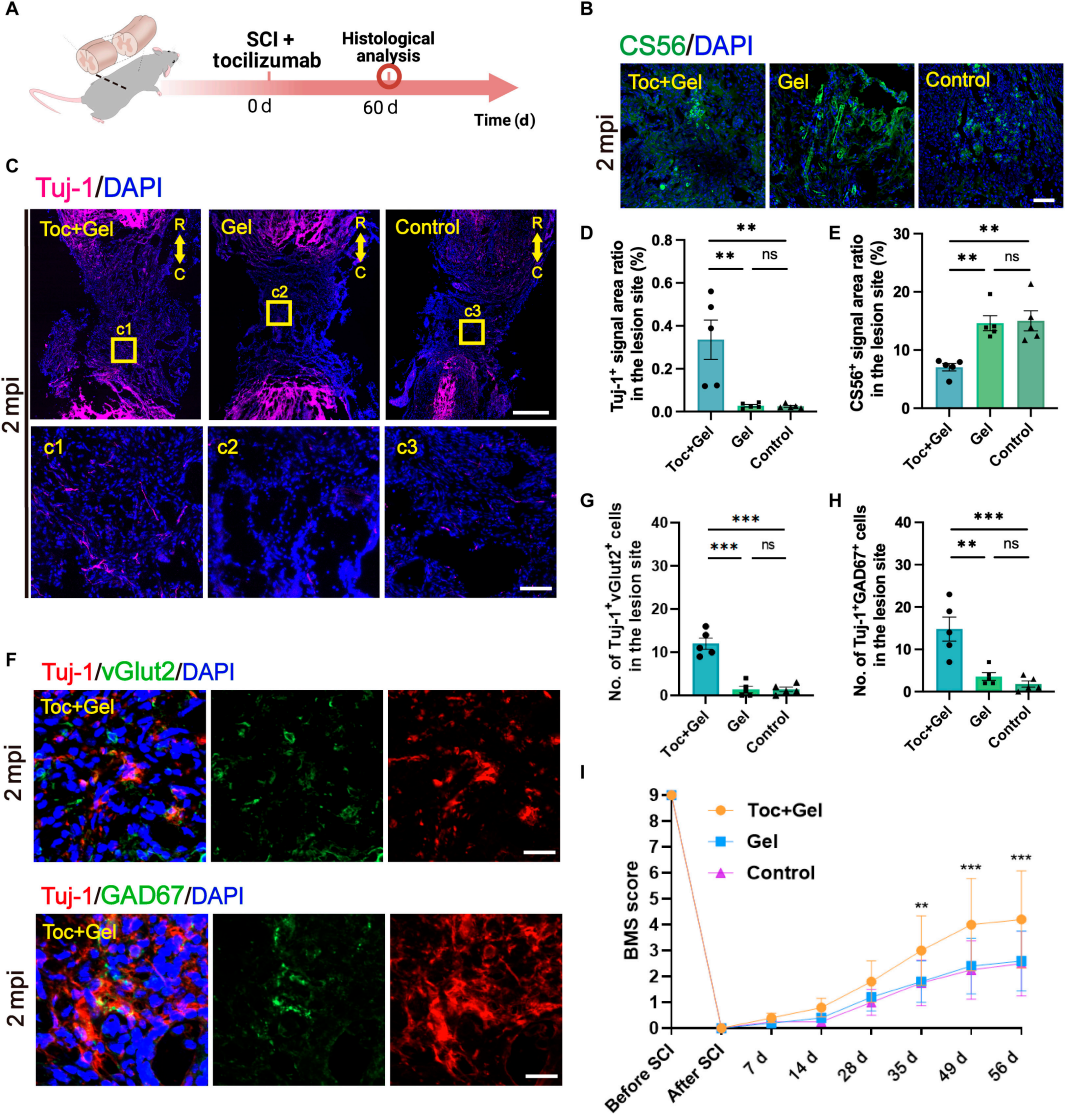
Tocilizumab supports prolonged axonal growth and motor function recovery in hind limbs. (A) Time point selection of spinal cord injury (SCI) model preparation and histological analysis. (B) The deposition of chondroitin sulfate proteoglycans within the lesion of 3 groups. The scale bar indicates 50 μm. (C) The profile of Tuj-1^+^ axons in 3 groups at 2 months postinjury (mpi). The long scale bar represents 200 μm, and the short scale bar represents 50 μm. (D) Statistical analysis of Tuj-1^+^ axonal area ratio per field in the lesion (*n* = 5 images). (E) The ratio of CS56^+^ area per field in the lesion (*n* = 5 images). (F) The expression profile of Tuj-1^+^ axons colabeled with vesicular glutamate transporter 2 (vGlut2) and glutamic acid decarboxylase 67 (GAD67) at 2 mpi. Scale bar = 50 μm. (G) Quantitative analysis of Tuj-1^+^vGlut2^+^ axons per visual field in 3 groups (*n* = 5 images). (H) The number of Tuj-1^+^GAD67^+^ axons per visual field in 3 groups (*n* = 5 images). (I) The mouse in the tocilizumab-treated group exhibited better hind limb locomotor performance in terms of Basso Mouse Scale (BMS) scores at 35 days postinjury (dpi) that persisted until 56 dpi. **P < 0.01; ***P < 0.001. Tuj-1, beta III tubulin‌.

In vivo analysis showed that tocilizumab improved the inflammatory microenvironment and promoted axonal regeneration in the early phase of SCI. We subsequently aimed to determine whether these early repair effects could yield long-term benefits and investigated changes in the regenerative microenvironment and axonal regeneration profile within the lesion site. The results showed that 2 months after early application of tocilizumab-loaded hydrogel, the deposition of chondroitin sulfate proteoglycans within the scar tissue decreased to around half the level detected in the Gel and Control groups (Toc+Gel: 7.031 ± 0.64; Gel: 14.61 ± 1.286; Control: 15.021 ± 1.724; *P* (Toc+Gel vs Gel) = 0.004, *P* (Toc+Gel vs Control) = 0.002; Fig. 8B and E). This suggests that early tocilizumab administration can attenuate long-term fibrotic scarring in the lesion and thereby enhance the regenerative microenvironment.

We further quantified axonal growth within the lesion and found an increase in Tuj-1^+^ axons in the tocilizumab-treated group, compared to those in both control groups, with axonal area coverage exceeding 0.3% (Toc+Gel: 0.335 ± 0.091%; *P* (Toc+Gel vs Gel) = 0.004, *P* (Toc+Gel vs Control) = 0.0037; Fig. 8A, C, and D). In addition, these Tuj-1^+^ axons colabeled with vGlut2 (an excitatory neurotransmitter marker) or GAD67 (an inhibitory marker), indicating the presence of functional axons. In contrast, the 2 control groups exhibited fewer Tuj-1^+^ axons and showed no difference between them (Fig. 8F to H). We also assessed the hind limb motor function of the 3 groups of mice with the BMS scores. Beginning at 35 dpi, the tocilizumab-treated group showed better motor performance compared to control groups (Fig. 8I). These findings indicate that early antibody intervention leads to the presence of more functional axons in lesions, forming the foundation for improved motor function recovery.

## Discussion

Inflammatory responses are inevitably triggered by SCI and represent a major driver of secondary damage, making them a long-standing target of neuroprotective strategies [15–21]. However, whether inflammation is ultimately beneficial or detrimental in central nervous system injury remains debated. Emerging evidence suggests that immune cells traditionally viewed as harmful, such as T cells and microglia, may also contribute to tissue repair [22–31]. Identifying key regulatory pathways and selectively modulating immune responses to maximize their reparative potential while minimizing cytotoxicity remains a challenge [32–34].

In previous studies, the IL-6R–JAK2–STAT3 pathway targeted by tocilizumab has been recognized as a potential regulatory hub, which not only is closely associated with the production of inflammatory factors but also plays an important role in axonal regeneration [17,35,36]. One team found that continuous intrathecal injection of tocilizumab reduces IL-6 secretion from M1 macrophages to restore tight junctions between vascular endothelial cells, thereby promoting SCI repair [37]. Although we also found that tocilizumab is generally therapeutic for SCI repair, the delivery system used in our study cannot specifically target the antibody to a single cell type. As antibodies are fragile and prone to inactivation during conjugation with bioactive materials, hindering their specific cell-targeting capability, future studies could design other drugs with single-cell-type-targeting functions to further dissect the effects of IL-6R blockade on resident cells in lesions. In addition, it is important to note that the pathophysiological peak of IL-6 mainly occurs at 1 dpi. Whether long-term application of tocilizumab or blockade of the IL-6 signaling pathway after IL-6 returns to normal levels would attenuate SCI repair remains to be discussed. If so, designing a reasonable therapeutic time window will be essential for the future clinical application of tocilizumab.

During the inflammatory cascade, necrotic cells release injury signals that recruit neutrophils and monocyte-derived macrophages while activating resident microglia, leading to the production of proinflammatory factors [38–44], including IL-1β, TNF-α, and IL-6—among the earliest up-regulated factors [45]. IL-6 has been shown to promote axon regeneration in previous studies [9,12,13], and this suggests that specific cytokines may participate in the repair process following SCI [46,47]. However, in our in vitro experiment, treatment of intrinsic spinal neurons to IL-6 or IL-6-pre-treated BV2-conditioned medium strongly reduced axon length, whereas IL-6R blockade restored axonal growth to baseline. These results contrast with earlier reports of IL-6-induced axonal regeneration in dorsal root ganglion neurons [12]. Whether this discrepancy derived from glial contamination during primary cell isolation or neuron-specific responses to IL-6 remains to be confirmed.

As the earliest responders to SCI, resident microglia play a crucial role in the inflammatory cascade [39,48–50]. After observing in vivo changes in glial cell polarization phenotypes, we performed RNA-seq on BV2 microglia in vitro. IL-6 treatment up-regulated immune-related membrane proteins and DEGs enriched in Th17 cell differentiation pathways. Although IL-6R blockade increased the expression of anti-inflammatory genes associated with NF-κB inhibition and IL-1 suppression, proinflammatory mediators such as matrix metalloproteinases remained elevated. These findings suggest that tocilizumab only partially suppresses microglial activation. Whether tocilizumab promotes tissue repair through microglia-mediated cross talk with other immune cells demands further investigation.

## Data Availability

Raw RNA-seq data have been deposited in the National Center for Biotechnology Information Sequence Read Archive under accession PRJNA1320693.
