# Supplementary material for: Targeting Interleukin-6 Signaling with Reactive-Oxygen-Species-Responsive Hydrogel to Promote Regeneration after Spinal Cord Injury
Source: Biomater Res. 2026 Jul 22;30:0395. doi: 10.34133/bmr.0395 (PMC13389045; doi:10.34133/bmr.0395)
Supplement: Supplementary 1 — Figs. S1 to S6 [file bmr.0395.f1.docx]

Supporting Information

**Targeting IL-6 signaling with Reactive Oxygen Species-Responsive Hydrogel to Promote Regeneration after Spinal Cord Injury**

Runlin Wen^2,1^, Xinghui He^2^, Kai Zhang^2^, Wanrong Ma^1^, Zhiquan Yang^2^ Dingyang Liu^2^, Ge Long^3^**^*^**, Xing Li^1^**^*^**

The Supporting Information includes Figure S1 to S6 for multiple supplementary figures.

**
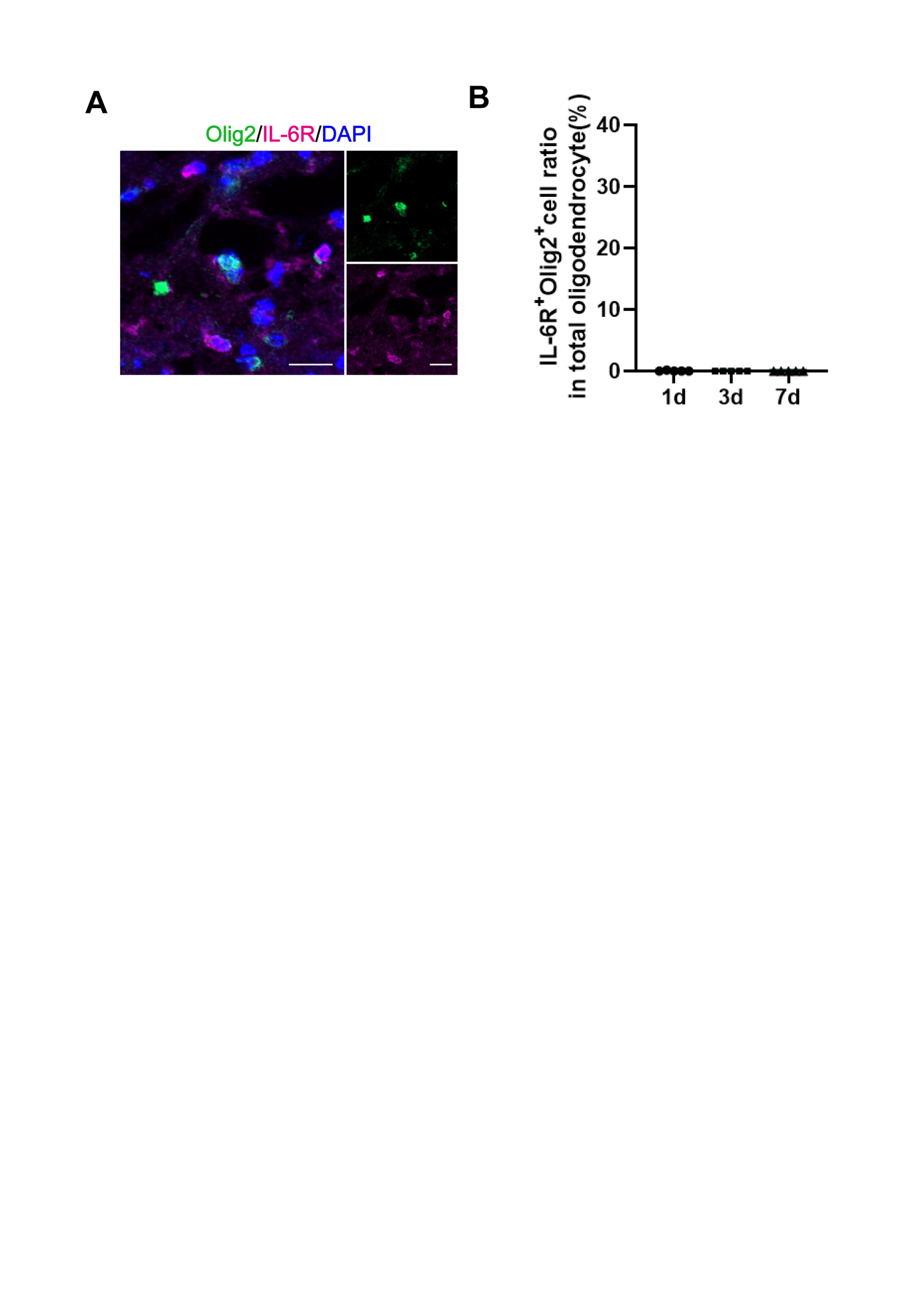
Supplementary Figures**

**Figure S1. The expression pattern of IL-6R in oligodendrocyte.** (**A**) No IL-6R expression is detected in oligodendrocytes at the core or margin of the lesion after SCI. Scale bar (short)= 20 μm. Scale bar (long)= 20 μm. (**B**) The proportions of IL-6R^+^ cells in oligodendrocyte (n = 5 images).


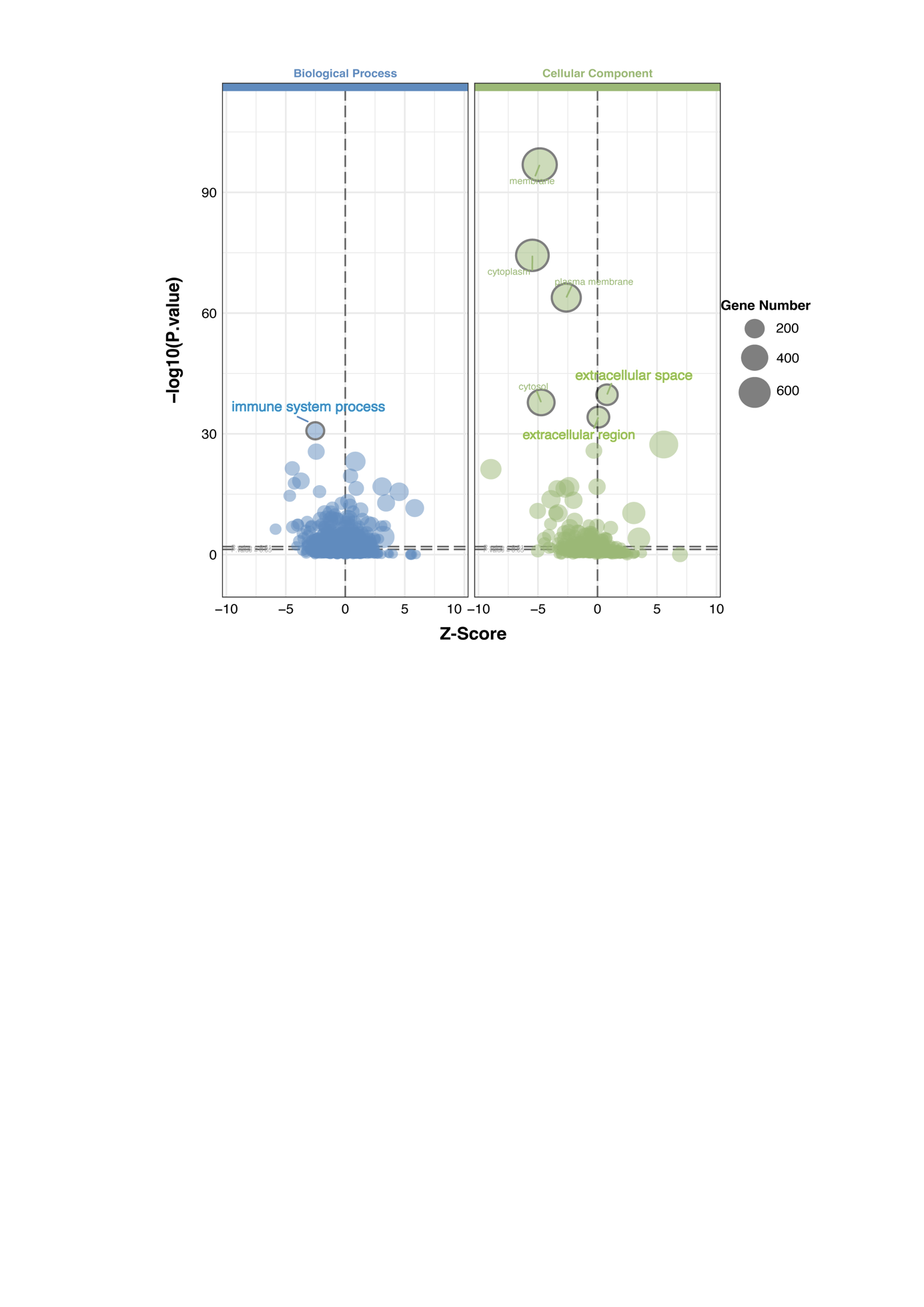


**Figure S2. Gene Ontology (GO) Enrichment analysis between the BVILToc and the BVIL group.** The most significantly enriched terms include the biological process "immune system process" and cellular component terms such as membrane, cytoplasm, plasma membrane, extracellular space, and cytoplasmic region.

**
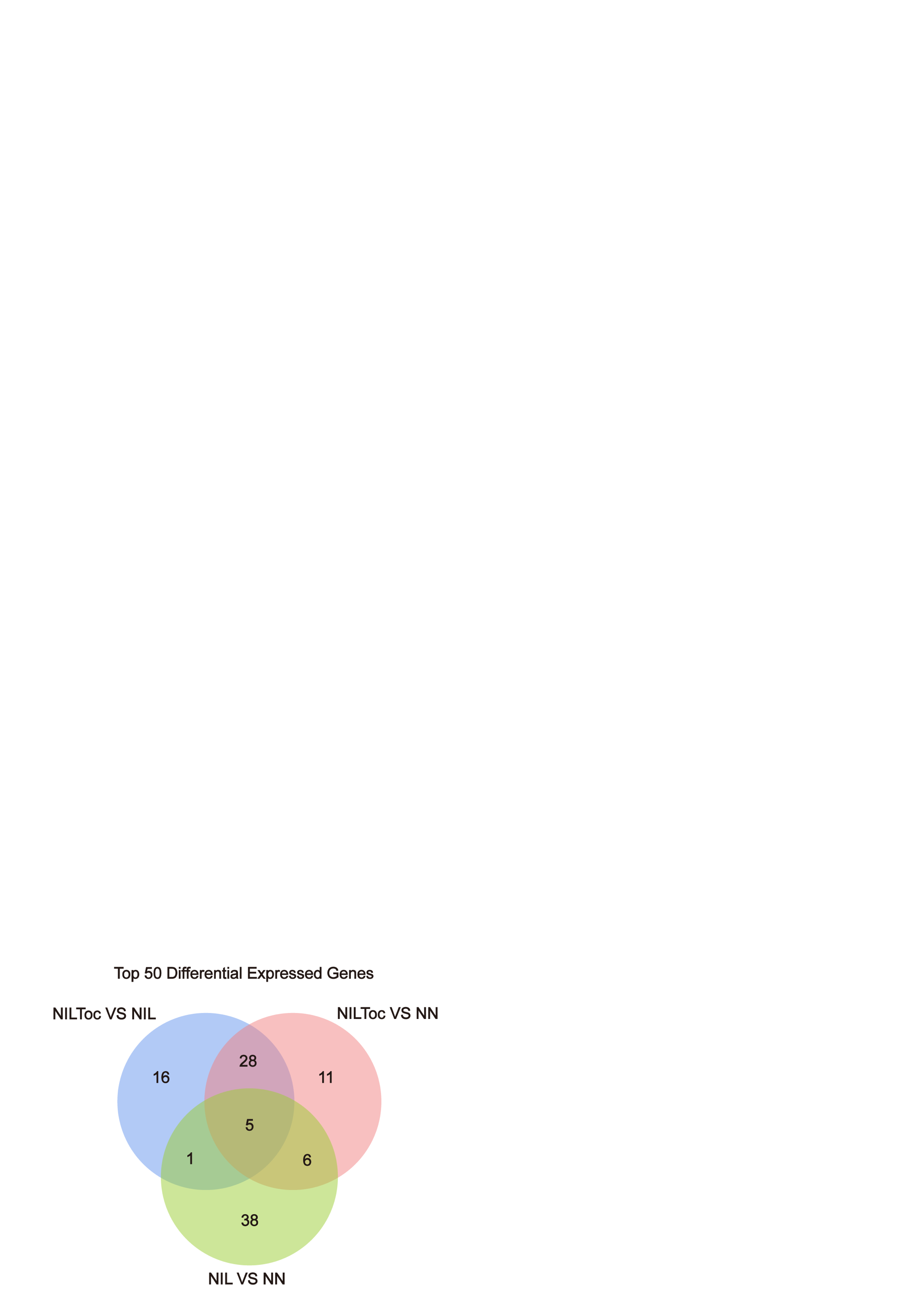
**

**Figure S3 Venn diagram of the top 50 differentially expressed genes among the three groups.**


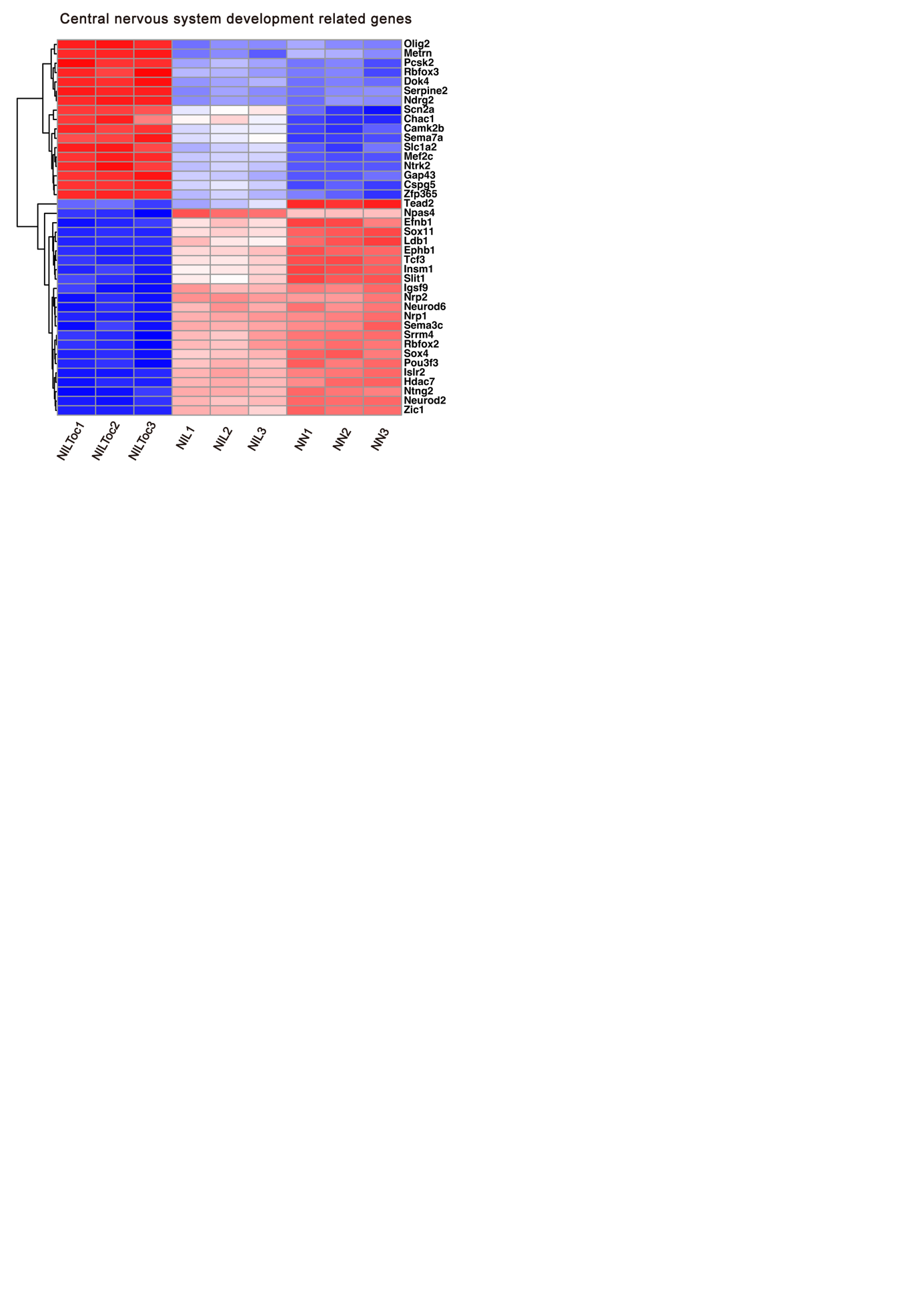


**Figure S4. Heatmaps of the top 40 differentially expressed genes associated with central nervous system development GO terms.**

**
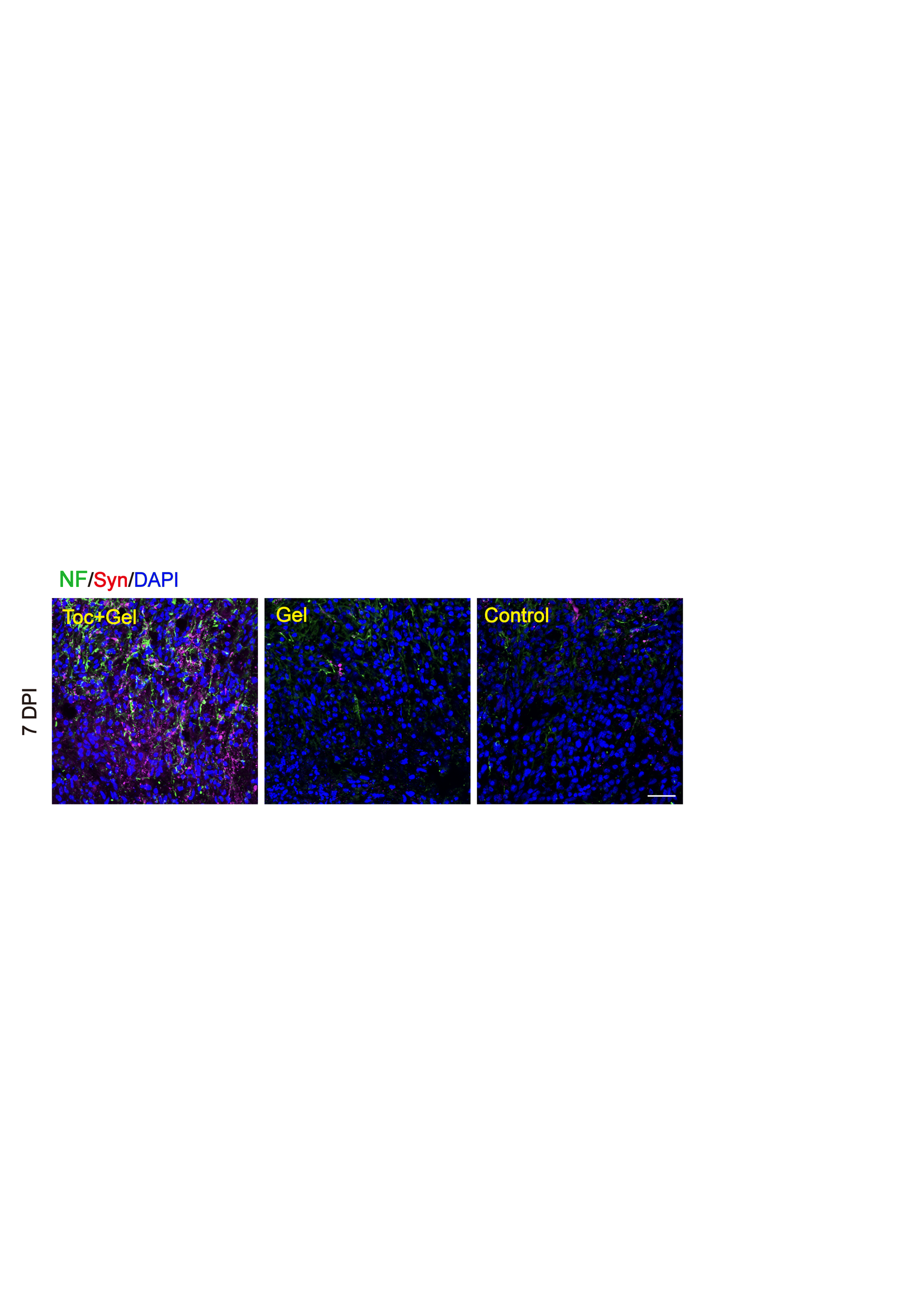
**

**Figure S5. Tocilizumab improves early axon regeneration and synapse formation at the lesion margin.** At 7 days after implantation of the Tocilizumab-loaded sustained-release hydrogel, there were more NF⁺ nerve fibers at the lesion margin compared with the control, and the synaptic protein Syn formed on their surface. Scale bar = 50 μm.


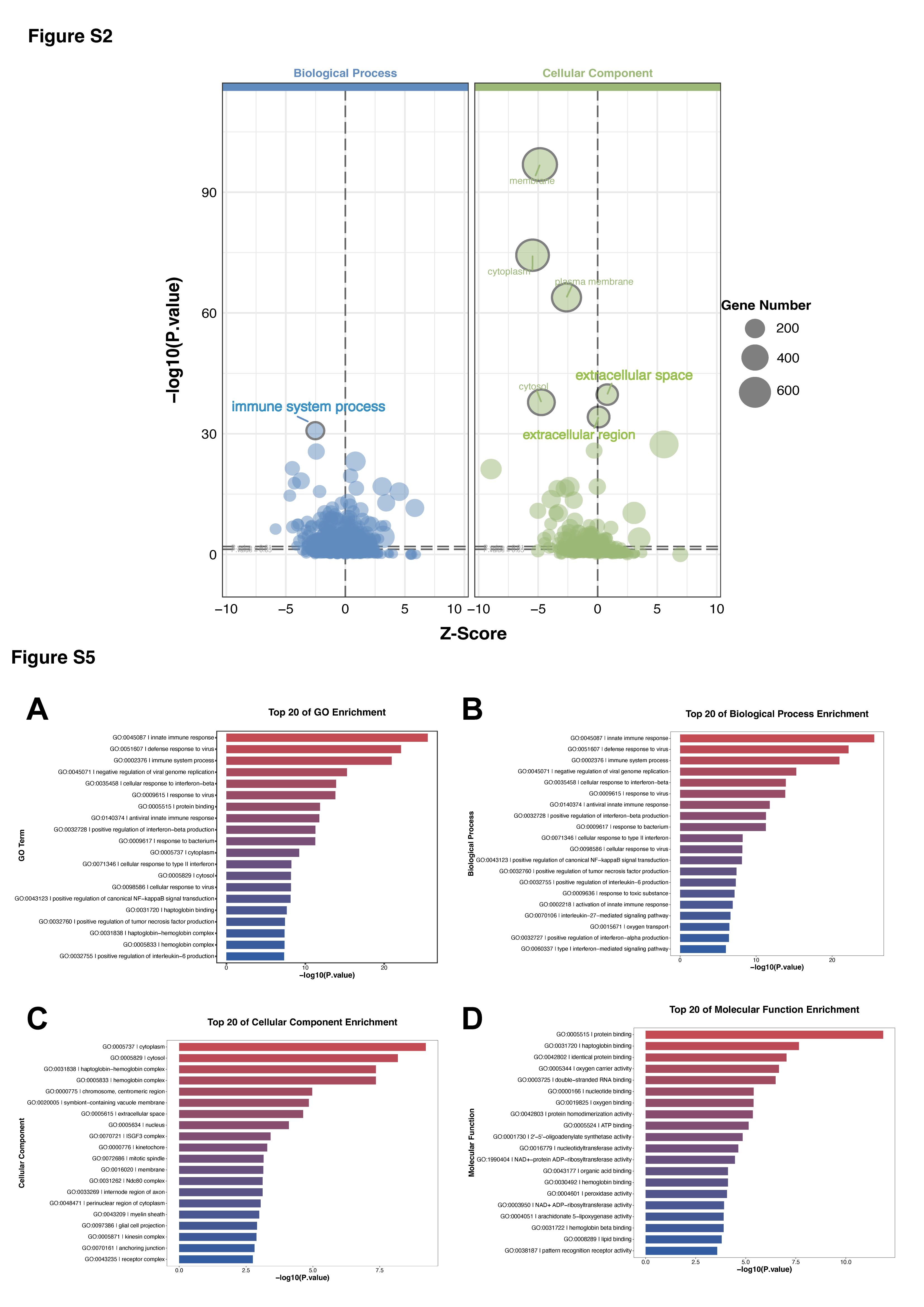
**Figure S6. Top 20 GO enrichment terms across three categories between the NIL and NN groups.**
